# The Art of Caring in the Treatment of Thoracic Outlet Syndrome

**DOI:** 10.3390/diagnostics8020035

**Published:** 2018-05-19

**Authors:** Julie Ann Freischlag

**Affiliations:** Wake Forest Baptist Medical Center, Wake Forest School of Medicine, Winston-Salem, 27157 NC, USA; jfreisch@wakehealth.edu

Those who diagnose and treat patients with thoracic outlet syndrome, especially those patients with neurogenic thoracic outlet syndrome, have a practice, which needs to include many modalities to diagnose, treat, and intervene to improve their quality of life for the present and for the future. Three key points constitute the mainstay of the art of caring for thoracic outlet patients. Initially, the most important thing is to make an accurate diagnosis. The second most important thing is not to offer interventions that will not help, perhaps harm, and to give false hope to those who have complex symptoms and have had interventions elsewhere without success. The third thing is to develop an algorithm of consistent evaluation and treatment for each patient to ensure an optimal outcome.

Maintaining a registry of your patients to truly understand your results and failures is essential. It is only by recognizing how your patients have done over time do you learn how best to take care of the next patient who seeks your help. We have learned that those neurogenic patients who are over the age of 40, and have had a dependency on narcotics for a long period of time, have other issues such as cervical disc disease, shoulder issues, and have a negative scalene block do not do as well with surgical intervention [1,2]. Additionally, those who undergo first rib resection and anterior scalenectomy, and never improve, usually fail to improve with a second operation. Whereas those who initially improve and then have recurrent symptoms about a year later with or without a history of repeat injury, can be treated with physical therapy and or Botox injections with great success [3]. Additionally, we know that about 50% of patients who have undergone first rib resection and anterior scalenectomy for venous thrombosis will have significant residual stenosis, which requires intervention to prevent recurrent thrombosis [4].

These 10 monographs offer the following salient points to improve your care of the patient with thoracic outlet syndrome:

Weaver and Lum summarize the new diagnostic and treatment modalities for those patients with neurogenic thoracic outlet syndrome [5]. They review the imaging techniques of computed tomography (CT) and magnetic resonance imaging (MRI) along with the value of median antebrachial cutaneous nerve (MABC) sensory nerve action potentials in identifying impingement if the brachial plexus. Updates in surgical techniques are reviewed including robotic and endoscopic approaches.

The importance of selecting a protocol in the treatment of vascular thoracic outlet syndrome is discussed by Archie and Rigberg [6]. They described their protocols in the treatment of both venous and arterial thoracic outlet syndrome. The treatment of both acute and chronic venous thrombosis, along with acute thrombosis, claudication, and asymptomatic arterial presentations, are delineated with excellent case report examples.

Humphries outlines the scope of a thoracic outlet syndrome registry and points out the important data to collect [7]. She also described how the combination of multiple registries in the future can play a role in the treatment of a condition like thoracic outlet syndrome due to the fact that many practitioners do not see a large number of patients.

Choosing the correct treatment, the correct timing of the treatment, and the succession to a different treatment for the patient with neurogenic thoracic outlet syndrome is the key for long-term success in alleviating symptoms for these patients [8]. These authors emphasize the need for a complete history and physical exam which will lead to the right intervention. They also outline the mainstay of the appropriate physical therapy protocol and the use of anterior scalene blocks.

The use of ultrasound in identifying anatomic variants in patients with thoracic outlet are described in detail by Leonhard and colleagues [9]. Utilizing both cadaver necks (82) and student subjects (22), brachial plexus variation was seen in 62.1% and 21%, respectively. Of the students, 50% had neurogenic thoracic outlet symptoms, which was higher than those with classic anatomy (14%). Ultrasonography can be helpful in diagnosis of neurogenic thoracic outlet syndrome, especially if provocative testing is negative.

The diagnosis and treatment of pectoralis minor syndrome is discussed in detail by Sanders and Annest [10]. This anatomical variant of thoracic outlet syndrome is rare but can be differentiated from neurogenic thoracic outlet syndrome by symptoms and physical exam, especially tenderness found in the axillary area. A pectoralis minor block can be used similarly to an anterior scalene block to make the diagnosis.

Utilizing a patient-centered care appraisal regarding symptoms before and after first rib resection, Ryan and colleagues tailor their diagnostic tests and intervention in patients with venous compression (McCleary’s syndrome) or venous thrombosis [11]. Their findings in 59 patients, who underwent first rib resection and anterior scalenectomy, demonstrated no difference in outcome if the patient had received thrombolysis, or when the rib resection had been performed which matched similar findings by Guzzo and colleagues [12]. Their conclusion is that paying attention to patient symptoms and not just vein patency can lead to appropriate intervention in patients with venous thoracic outlet syndrome.

Peek and colleagues report on a retrospective multicenter study on patients who underwent operations for thoracic outlet syndrome from 2005 to 2016 [13]. Patients were assessed by the 11 item version of the QuickDASH questionnaire. Sixty-two patients were evaluated—36 neurogenic, 13 arterial, 7 venous, and 6 combined—and 73% returned the survey. Fifty-four percent (27) had complete relief and 90% had improvement. These findings were similar to previous findings by Chang [14] and Rochlin [15], when patients are chosen appropriately.

A unique report on high performance musicians who played bowed string instruments is presented by Adam and colleagues [16]. Sixty-four high performance musicians were evaluated and compared to 52 healthy volunteers with duplex scanning and provocative maneuvers. Duplex scans were abnormal in 69% of musicians showing compression, as compared to 15% of controls (*p* = 0.03), and provocative maneuvers were positive in 44% of musicians as compared to 3% of controls (*p* = 0.03). This alerts us to the high incidence of potential thoracic outlet syndrome in these musicians as many of us has seen and treated them.

An excellent summary of the present state of the art of diagnosis, treatment, and outcomes is presented by Povlsen and Polvsen [17]. They hypothesize that the ability to stratify patients according to their exact compressive mechanism could lead to better outcomes.

In summary, these 10 informative manuscripts provide a roadmap for the future excellent treatment of those patients with thoracic outlet syndrome.
